# Abstracts and Gleanings

**Published:** 1887-06

**Authors:** 


### ABSTRACTS AND GLEANINGS.

**Treatment of Hemorrhagic Malarial Fever.**—In the milder forms of the disease, this is simple, easy, and successful, as already illustrated in the cited cases of Drs Bemiss and Harley, a few doses of calomel, followed with quinine, being all that was necessary. When, however, the type is violent and malignant, we will have ample scope for the exercise of our deepest thought and grandest skill. In these malignant cases the patient will be troubled from the onset with distressing nausea, frequent retching and vomiting of a greenish fluid with mucous flocculi, the pulse will be small, frequent and feeble. the skin shriveled and surface cool or cold, with shrunken and pinched features, and the frequent emission of dark, bloody urine, presenting the appearance of disintegrated and broken down red-blood cells, loaded with the bile pigments in the form of small black granules. In all such cases the urine when carefully examined with the microscope will be found almost, if not entirely, without a single red-blood corpuscle. These are the severer types of this disease, most malignant in character, that claim our attention. When called to a case of this type we should promptly apply dry cups to the epigastrium and right hypocondrium, with a view to derive the blood from the congested vessels of the stomach and liver to the cutaneous surface, and follow the cups by a blistering plaster over the same regions to maintain the circulation in the skin and to aid in stimulating the liver to its secretory action. Give hypodermic injections of morphia to quiet nervous irritation and to compose the stomach to rest. Give to adults 5 grains of calomel with 3 grains of bi-carb. soda every three to four hours till the alvine discharges show the presence of bile, and the liver again resumes its work; after three or four doses have been given, if the bowels have not been moved, give enemas of plain warm water, or containing a desertspoonful of camphorated oil, or a small teaspoonful of table salt. If the stomach should still be troubled with nausea or tendency that way, give an emulsion, each dose consisting of 1 drop creasote, 1-12 to $\frac{1}{8}$ sulph. morphia, 3 grains bi-carb. sod., and a drachm aqua menthæ, and repeat every two to three hours as long as required. Frictions over the back with dry mustard and pulv. capsicum, as also to the arms and legs, should be assiduously applied, supplemented with dry heat to the body. As soon as the calomel has produced a well-marked bilious fecal discharge, or reaction is manifest, no time should be lost in pushing quinine, but not in doses too large. It is all a mistake and highly mischievous to give very large doses of quinine, except in the congestive pernicious forms of intermittent fever, where it is an absolute necessity to prevent the recurrence of another paroxysm. But in this disease, with this type, 5 to 10 grains every three to five hours, till there is slight manifestations of cinchonism, and then at longer intervals, so as to keep up a moderate impression, for the purpose of antagonizing the malarial poison and giving tone to the nervous centers.

The quinine, if the stomach will not tolerate it, should be given hypodermatically, which is the preferable plan, on account of the danger of again setting up nausea and vomiting. As soon as the function of the liver is measurably or appreciably established, and enough quinine has been introduced into the system to make its physiological effects manifest, commence to give the muriated tinct. of iron in full doses, 20 to 30 drops every four hours, with a view of toning up the debilitated blood-vessels, reconstructing and vitalizing the hæmoglobin and the red-blood corpuscles, and thus restoring the normal and vital condition of the blood. If the kidneys should fail to excrete urine, use frequent frictions over the lumbar region with warm whisky, spts. turpentine and tinct. digitalis, which will often be found efficient in stimulating the kidneys; indeed it will always be found useful both in stimulating the kidneys and acting favorably upon the mucous membranes, both of the stomach and bowels and of the urinary passages.

I have now given in detail my convictions of the etiology and essential nature of this disease, together with my plan of treatment and the medicines used, which I would recommend as being fairly successful—certainly as much so, if not more, than under other and diverse plans which I have seen others practice—and withal it is so rational and fulfills so completely every pathological condition and indication of treatment that even when not successful, for some must of necessity die, that the physician's mind is left with the composing reflection that he could have done nothing more to turn away the shafts of death and bring his patient back to health again.

I have concluded my paper without any reference to the dietary. Every physician will know that with such a broken-down state of the blood, and such a debilitated and impoverished condition of the digestive and assimilating organs, that the necessity for feeding is great, but that only the most nutritious and most easily digested and assimilable foods should be allowed, and they given in small portions, but often repeated, increasing the quantity, *pari passu*, with the improving strength.

As soon as the patient will bear moving, a dry, bracing atmosphere and a healthy location should be sought, and the patient advised not to return to his malarious home, for we should never forget the pertinacity of the poison. Says Dr. Harley, "In fact, the poison of the worst forms of malaria seems to saturate the tissues and adhere to the constitution with as much tenacity as the poison of syphilis; for there is no period of an individual's life, after he has once had a bad attack of malaria, at which he may be said to have completely gotten over it."—*Dr. Day in La. Medical Society.*

**Collodion of Iodoform** has been successfully used for the relief of neuralgia, and is usually prepared by dissolving 1 part of iodoform in 15 parts of collodion. Occasionally 10 per cent., and even 25 per cent. solutions have been employed.—*Nouv. Remedes*, 1886, p. 525. An older formula by James directs iodoform 5, balsam of Peru 5, powdered soap 5, and collodion 85 parts.

**The Subiodide of Bismuth in the Treatment of Ulcerations.**—The subiodide of bismuth unites the properties of iodine and bismuth; the former acts upon granulation tissues, inducing an increase of all normal physiological processes, producing a protoplasmic energy ordinarily obtained only by congestion, hyperæmia or inflammation, and induces a reversion of the tissues to an embryonic state; it imparts a formative influence or force to the cellular elements of the three germinal or blastodermic layers and their derivatives, closely approximating the laws of normal development; its specific action in certain dyscrasiæ and its antiseptic power, inhibiting germ development in the proportion of 1 to 4,000 add to its efficiency. Bismuth unites with the albumen, forming an albuminate, which acts as a sedative, aseptic, impermeable coating on the granulations, retarding the evaporation of the cellular moisture, modifying the formation of a striated connective tissue from the gelatinous intercellular substance, and prevents the retrograde metamorphosis of the white blood corpuscles. Summarizing the physical and therapeutic properties of the subiodide, it may be said that,

1. It subdues the inflammatory process.
2. It substitutes the inflammatory regeneration of tissue, with a neoplastic force, which closely simulates normal embryonic histogenesis.
3. It is by its action a vital antiseptic, supporting life, promoting nutrition and maintaining the integrity of the tissues.
4. It prevents the formation of pus.
5. It lessens direct and reflex irritability.
6. It is so bland and unirritating that it can be used on any surface.

Under the use of subiodide of bismuth, ulcers, which usually heal by cicatrization at the rate of one-half to one line per da,y have been seen to dermatize at the rate of three to five lines per day; the cicatrix is fleshy and firm, and is but little disposed to destructive inflammations or subsequent alterations even over syphilitic sores. Certain ulcers of particular origin may require some radical or specific treatment to convert them to simple forms of ulcer or to remove pathological elements, which would retard healthy tissue formation. The dressing is applied as a dry powder.—*Dr. Reynolds in Medical News.*

**Triple Contagion of Tuberculosis—from Man to Man, from Man to Fowls, and from Fowls to Man.**—Dr. de Lamaileree publishes, in the *Gazette Medicale de Paris*, the following interesting facts: The little village of C. is a most healthy spot, being about 2,000 feet above the level of the sea. Epidemics are there unknown, and the inhabitants of this village generally die of old age or pneumonia. In 1872 a young man who had contracted bronchitis whilst a prisoner in Germany during the war, settled down in this village. He married a strong, healthy girl; soon afterwards he began to spit blood, and died of consumption two months after the birth of a son, and within a year of his marriage Soon after his death his wife, who had nursed him, had bronchitis, which became chronic, and in a little while tuberculosis of the lungs was manifested. The child had successive attacks of bronchitis, and rapidly developed consumption. Cavities formed in the woman's lungs, and she expectorated abundantly. A short time ago the physician attending her was called to a young woman in the same village who showed evident signs of pulmonary phthisis. The house was at some distance from that of the first female patient. The second patient was a woman, aged twenty-nine, and of a robust constitution. A careful examination revealed that she rarely went to the house of her neighbor, who had contracted consumption, and never ate or slept there, but that she had eaten the flesh of eleven fowls which had died at her invalid neighbor's during the space of four months. She had eaten them very underdone, believing that they were most nourishing when but slightly cooked. It was discovered that these fowls had swallowed some of the sputa expectorated by the first patient. The birds had been seen to collect round her whenever she coughed. On making a necropsy of one of the fowls which had just died, it was found that the intestines and liver were filled with tubercles. The fowl had become very emaciated, and could hardly move; the purulent liquid found in the tubercules contained in the liver was filled with bacilli tuberculosis. It was probable that the other birds had perished from the same cause. These fowls must, therefore, have been the means of conveying the contagion to the second woman who had eaten them. In this case there is the triple contagion of tuberculosis.

1. From man to man.
2. From men to animals.
3. From animals to men.

Contagion from man to man is already a scientific fact. Contagion from men to animals had been admitted by many writers, but others have stated that certain animals, amongst which are fowls, could not be inoculated. This case shows clearly that fowls are as liable to contract tuberculosis as other animals, such as cats and dogs. Contagion from animals to men is clearly demonstrated in this case. Up to this time the only known vehicle of contagion was cow's milk; now it is shown that the bacillus can also be carried through fowls. It is important, then, to pay great attention to the health of fowls, destined for food, and it would be worth while to find out how soon after the fowl has begun to suffer from tuberculosis it can infect those eating it, and also how much cooking will destroy the bacilli.—*British Medical Journal.*

**Measles.**—This winter measles has prevailed very extensively, and we are likely to have a season of it. It is not worth while to say anything about diagnosis, for this is one disease in which diagnosis is clear; but I am not so sure about the treatment. Certainly our *regular* neighbors have a most vicious treatment.

I will say first that measles can be successfully treated with *hot water* alone. Let it be drank freely, and if the patient complains of any point of pain, let it be sponged with hot water and covered with dry hot flannel.

My medication of measles is somewhat stereotyped, but it is very satisfactory. To a half glass of water add a half-teaspoonful of asclepias and five drops of droseria, and give a teaspoonful every hour.

When there is the slightest duskiness of surface, alternate the above with baptisia. I do not think we would hear of so many deaths from "*black* measles," if baptisia was used.—*Cincinnati Eclectic Medical Journal.*

**Dangers of Santonin.**—Dr. Laure, at a recent meeting of the Lyons Medical Society, related an interesting case of santonin-poisoning. On December 25, last, he was called to visit a child three-and-a half years old. The patient was lying on his back, in a state of deep prostration, now and then interrupted by sharp cries. The child then would bend up its knees, and place its hands on its abdomen, apparently the seat of the pain. This fit over, he would fall again into the former somnolence. The face was of a livid paleness, the pupils dilated, breathing frequent, the pulse rapid and irregular, while the rectal temperature remained below 37° C. (98.6° F.). The stomach would immediately reject anything ingested.

As regards the dose of santonin, Dr. Laure thinks the rule laid down by Benzinger, viz , as many grains as the child has years, four days in succession, is one that should be regarded with suspicion. He prefers Wood's advice—not to exceed one grain for a child less than two years. Dr. Laure is also of opinion that it is very desirable to combine santonin with a purgative; calomel, for instance. As to the antidotes of santonin, ether, and especially chloral, have been recommended as the best by Becker and Binz. In a case when convulsions or nervous troubles occur, they would have been used willingly. But in the case just narrated it was thought more advisable to facilitate the elimination of the poison by the kidneys and bowels, and afterwards to administer tonics.—*Therapeutic Gazette.*

**A Speedy Cure of Whooping Cough.**—Mohn, a Norwegian physician, had in his own family a case of scarlatina and whooping-cough. After the violence of the scarlatinal attack had subsided, but while the whooping-cough was still present, Mohn disinfected the child's bedding with the fumes of sulphur. Just previous to the fumigation the child had a severe paroxysm of coughing, which led the father to hesitate in his employment of the sulphur. He was surprised and gratified, however, to observe that the disease was cured ; and the patient's sister, who had a cough, the sequela of pertusis, was also cured. These children were not included in the fumigation intentionally, but inhaled such vapor as casually permeated their apartment.

In a subsequent severe case, the writer, after the failure of other means, had recourse again to inhalations of sulphur gas, with prompt success. He cites successful cases of five months in age, and children of varying ages have been cured in this manner. He proceeds as follows: The patients are dressed in clean linen, and taken from their bedroom to another room. In their absence bedding, furniture, playthings, linen, clothing, everything which the sick-room contained is so arranged that the fumes of sulphur can penetrate to all. Six and a half drachms of sulphur per cubic metre of air-space in the room are burned, and the fumes allowed to permeate the room for five hours. At evening the child is taken back to the sick-room and put in a bed which was disinfected; it awakes the next morning cured.—*Revue Internationale.*

**Cold Bathing.**—*The Lancet* says the use of cold water as a bath for ordinary health purposes—we are not speaking of its use for the strictly medical purposes of reducing the temperature of the body in certain states of diseases—is purely reactionary. The cold bath is only useful, or even safe, when it produces a rapid return of blood to the surface immediately after the first impression made, whether by immersion or affusion. The surface must quickly redden, and there must be a glow of heat. If these effects are not rapidly apparent, cold bathing is bad; and no such effects are likely to be produced unless the circulation be vigorous and both the heart and blood vessels are healthy. Great mistakes are made, and serious risks are often incurred, by the unintelligent use of the cold bath by the weakly or unsound. Moreover, it is necessary to bear in mind that there is seldom too much energy to spare after middle age, and it is seldom expedient for persons much over forty to risk cold bathing. We would go so far as to say that no one above that age should use the tub quite cold unless under medical advice. It is possible to be apparently robust and, for all the average purposes of life, healthy, and yet to have such disabilities arising out of organic disease or weakness as to render the recourse to heroic measures, even in the matter of cold bathing, perilous.—*Maryland Medical Journal.*

**Point of Diagnosis in Rotheln.**—In the *Lancet*, April, 1886, p. 785, Dr. Glover says he has noticed the earliest symptom to excite notice in cases of rotheln or German measles, is a swollen gland in the neck at the back of sterno-mastoid muscle. This symptom he has noticed four or five days before the rash appears. When disease is prevalent, or already exists in a family, and a swollen cervical gland in a young person appears, without obvious reason, it may be suspected that the system is already infected.—*Southern Practitioner.*

**Management of Simple Constipation.**—Sir Andrew Clark gives the following instructions for the management of simple constipation:

1. On first waking in the morning, and also on going to bed at night, sip slowly from a quarter to half a pint of water, cold or hot.

2. On rising, take a cold or tepid sponge-bath followed by a brisk general toweling.

3. Clothe warmly and loosely; see that there is no constriction about the waist.

4. Take three simple but liberal meals daily; and, if desired, and it does not disagree, take also a slice of bread and butter and a cup of tea in the afternoon. When the tea is used it should not be hot or strong, or infused over five minutes. Avoid pickles, spices, curries, salted or otherwise preserved provisions, pies, pastry, cheese, jams, dried fruits, nuts, all coarse, hard, and indigestible foods taken with a view of moving the bowels, strong tea, and much hot liquid of any kind, with meals.

5. Walk at least half an hour twice daily.

6. Avoid sitting and working long in such a position as will compress or constrict the bowels.

7. Solicit the action of the bowels every day after breakfast, and be patient in soliciting. If you fail in procuring relief one day, wait until the following day when you will renew the solicitation at the appointed time. And if you fail the second day, you may, continuing the daily solicitation, wait until the fourth day, when assistance should be taken. The simplest and best will be a small enema of equal parts of olive oil and water. The action of this injection will be greatly helped by taking it with the hip raised, and by previously anointing the anus and the lower part of the rectum with vaseline or with oil.

8. If the use of drugs is unavoidable, try the aloin pill. Take one-half an hour before the last meal of the day, or just as much of one as will suffice to move the bowels in a natural way the next day after breakfast. If it should produce a very copious motion, or several more small motions, the pill is not acting aright; only a fourth, or even less, should be taken for a dose. When the right dose is found it may be taken daily, or on alternate days, until the habit of daily defecation is established. Then the dose of the pill should be slowly diminished, and eventually artificial help should be withdrawn. The aloin pill is thus composed:

℞. Aloinæ .................................. gr. ss,
Extr. nucis vom .......................... gr. ss,
Ferri sulph .............................. gr. ss,
Pulv. myrrhæ ............................ gr. ss,
Saponis .................................. gr. ss,
Fiat pil. i.

[We suggest *ipecac*, ss grain, in place of the *sulph. ferri* in the above pill.—ED. RECORD.]

If the aloin pill gripes, provokes the discharge of much mucus, or otherwise disagrees, substitute the fluid extract of cascara sagrada, and take from 5 to 20 drops in an ounce of water, either on retiring to bed or before dinner. And when neither aloin nor cascara agrees, you may succeed by taking before the midday meal 2 or 3 grains each of dried carbonate of soda and powdered rhubarb.

The exact agent employed for the relief of constipation is of much less importance than its mode of operation. If, whatever the agent may be, it succeeds in producing after the manner of nature one moderate formed stool, it may be, if necessary, continued indefinitely without fear of injurious effects. But treated upon physiological considerations, I have the belief that in the great majority of cases simple constipation may be overcome without recourse to aperients.—*London Lancet.*

**The Rational Treatment of Patients After Cataract Operatious.**—At the Chicago Society of Opthalmology (*Chicago Medical Journal*) Dr. F. C. Hotz read a paper on the subject:

Though the safety of the eye is of prime importance, we must, in our anxiety for the recovery of this organ, not entirely disregard the comfort of the patient, for we have no right to deprive our patients of any comfort which does not disturb the healing process. The usual treatment after cataract operations is anything but pleasant to the patient, and many dread the confinement to bed, and in a dark room, worse than the operation itself. If it can be shown that such measures are superfluous, they should be abandoned.

When a wound is carefully cleansed and thoroughly disinfected, and its edges are nicely approximated, it will heal by first union, provided the close adaptation of its edges is not disturbed by external violence or by muscular tractions. Rest of the wounded part is the first condition for kind healing. But to rest the eyeball it is not necessary to put the patient to bed; we can secure the necessary rest for the operated eye., i. e., we suspend the functions of the ocular muscles and immobilize the eyelids by bandaging *both* eyes. As long as they are closed they remain comparatively motionless. The bandage over both eyes accomplishes this result just as well whether the patient is in bed or sitting in a chair. The confinement to bed, therefore, is irrational, and the recumbent posture is not only of no advantage, but may even disturb the healing, because it favors the flow of blood to the head; consequently congestion of the ocular tissues is more likely to occur in the recumbent posture than when the patient is sitting up.

The uselessness of darkening the room will be apparent to everyone who will bandage his own eyes just in the same manner as we dress the eyes after cataract operations. The bandage shuts off the light so thoroughly that we will be unable to tell whether the room is light or dark. To the patient it is immaterial, but to his attendant it is a great comfort to have light in the room.

In regard to the dressing there is a difference of opinion: the majority of oculists employing the pads and bandage: some closing the eyelids only by strips of plaster, and others discarding all dressings.

If we dispense with the dark room, we cannot leave the eyes without dressing, because the winking of the eyelids and the constant rotations of the eyeball would disturb the wound and cause a good deal of irritation.

The plaster strips overcome this difficulty; but they do not give the eye any protection against mechanical insults which might cause a re-opening of the freshly united wound. The bandage with padding of absorbent cotton or any other soft material secures to the eye rest and protection against accidents and is therefore to be recommended as the most rational dressing. The flannel roller is open to the objection of being easily disarranged by the movements of the head on the pillow; but a roller of mosquito netting or Swiss gauze applied wet, forms, when dry, an immovable bandage which does not become loose, and keeps nicely adjusted for any length of time.

The sensitiveness of the operated eye to bright light is not due to the bandage; it varies greatly in different persons; some even do not show it at all. Where it exists it only shows that the eye has not completely recovered from the effects of the operation though the external wound may appear well healed. With the eyes well bandaged my patients are sitting in easy or rocking chairs, in light rooms, and are even allowed to take an exercising walk in their rooms; they go to bed when they please, and get up whenever they like. I have never observed any complication or accident which could directly or indirectly be attributed to this mode or treatment. The recovery was as rapid and the results as good as under the old-fashioned regime.

**Clergyman's Sore-Throat.**—Thos. Whipman, M. B. (*Lancet*, Sept. 4), in an article on this subject, compares the lawyer's and clergyman's method of delivery; he says, to ascertain what happens when the clergyman or the barrister is addressing an audience it is only necessary that one read aloud, holding his head erect as the barrister does, with his chin slightly elevated; and then continue to read in the same tone, but allow his head to fall forwards as the clergyman does, so that his head shall nearly rest on the sternum. The change in the voice will at once strike the listener who will straightway appreciate the muffled and indistinct tones which accompany the forward inclination of the reader's head. In two severe cases of the disease the writer applied glycerineboracis at first, and astringents subsequently, with rest, and a habit of holding up the head during delivery. Both patients expressed in strong terms their sense of "a speedy relief to their sufferings" owing to the ease and comfort with which their services were performed with the head in the natural position.—*N. E. Medical Monthly.*

**Treatment of Chronic Ulcers.**—Heidenhain says that the best method of dealing with old chronic ulcers, especially of the leg, is to dress them with a considerable thickness of absorbent cotton. The cotton is pressed upon the ulcers by a roller bandage, and is allowed to remain undisturbed until, after the lapse of five days or a week, the secretions come through. Then it will be found that delicate healthy granulations have sprung up in place of the dirty necrotic appearance erstwhile presented, and the torpid callous margins are considerably improved in appearance. The dressing is then re-applied and changed as before. The advantage of this method lies in its being absolutely painless. No septic infection need be feared from absorption of pus. The dressing remains sweet until it is so saturated that the discharge comes through, when a change should at once be made. By actual experiment, the superiority of this dressing over the method of compression by adhesive plaster strips has been demonstrated.

After the cotton dressings are no longer needed, the surface may be dressed with zinc ointment, after irrigation with carbolic acid; if more stimulation is desired, a 2½-per cent. iodoform ointment answers admirably. Grafting should be employed if the ulcer is of great extent.

In order that the new formed skin does not crack and break, when the limb is again put to use, it is advisable to oblige your patients to take some exercise during the process of repair. The ulcer does not heal so quickly as if absolute rest be observed, but the result is a more permanent one. In such cases that are obliged to be a bed on account of the large size of the ulcer, Heidenhain has found it of great advantage to bandage the legs in a flexed position. Thus the skin and the soft parts are kept at a certain tension during the healing process. If the limb be kept at rest fully extended, the cicatrix will surely tear open when walking is resumed. To keep the leg flexed, the use of a double inclined plane is very serviceable.—*Weekly Medical Review.*

**The Cure of Large Ulcers of the Leg by the Carbolized Spray.**—Giles de la Tourette,[t] of Paris, presents in detail three cases of stubborn ulcers of the leg in inmates of the *Infirmery des Incurables* connected with the service of Charcot. The general condition of the patients was of the worst description. The first was an extremely debilitated and emaciated subject of asthma conjoined with chronic bronchitis, sixty-nine years old, presenting an enormous ulcer covering the whole right leg, from the malleoli to within an inch of the tuberosity of the tibia. The carbolic spray for an hour and a half morning and evening, with intermediate dressing with borated vaseline, caused a complete cure in less than a month. The second was an ulcer, eighteen centimeters high, covering the lower half of the leg in a patient eighty two years of age, feeble and senile, and the subject of chronic bronchitis. Carbolized spray for two hours twice a day with borated vaseline dressing in the intervals, secured a complete cure in about six weeks. The third case occurred in a syphilitic subject, with mitral insufficiency, tertiary syphilis and chronic bronchitis, aged fifty nine, with a vast ulcer, twenty-two centimeters long, enveloping the whole leg. Carbolic spray applied during the next six months had brought the ulcer down to about the size of a six sous piece when the patient died by his own hand. As the result of his observations, the author concludes:

1. The method of carbolic spray repeated daily for an hour and a half, morning and evening, better than any other method leads to a rapid cure of large varicose ulcers.

2. In the early part of the treatment, the pains seated in the ulcer disappear. In the three cases observed, no erythema ever appeared at the margin of the wound, nor did the patient ever void the black urine indicating carbolic poisoning.

3. A state of debility or senilty of the patient does not in any way contra-indicate the employment of the method, which, on the contrary, by the local stimulation, which it determines, seems formally indicated in this particular case.

4. The solutions used are the stronger, as the ulcer is the more atonic; solutions less than 1 to 50 should be rejected and even the greater strength, 1-30, 1-20, or even 1-10 could be used. In the interval between the applications of the spray, a dressing of borate of soda and vaseline, 1 to 10 will be found useful.—*Revue de Chirurgie, July, 1886.—Annals of Surgery, March, 1887.*

**Inoculation as a Preventive of Yellow Fever.**—Dr. Bustamente, of Cucuta, Colombia, says of this subject: "Dr. Urricoechea, surgeon of the frontier battalion, inoculated, by way of experiment, and with good results, five of his soldiers. Twenty minutes after the operation the temperature gradually ascended to 40°C., accompanied with all the symptoms of yel ow fever. This lasted forty hours, at the expiration of which had disappeared the fe\er and all attendant symptoms. This operation was effected in a place called Moras, three leagues from Cucuta, and where a body of troops were stationed, who have not come to this city for fear of the fever. At present the inoculated soldiers are here, exposed to the action of the focus of infection. As in Moras, no case of the epidemic has as yet presented itself." Dr. Bustamente, in his letter, states that "as my labors in the field of inoculation, as a preventive of yellow fever are only, it may be said, mere experiments, which, although they may satisfy me with a well-founded hope of successful and complete result, cannot be of genuine utility until the best and most efficacious method is decided upon. I am thinking, however, of making an abstract of my observations, together with the method pursued, the results obtained, and everything that may be useful in the premises. For the present I will confine myself to the statement that in more than forty persons whom I have inoculated, a fever, with many of the characteristic symptoms of yellow fever, has presented itself; this fever, developed by inoculation, varying several tenths of a degree, and in some cases ascending to 41° C., but never presenting the most grave symptoms of yellow fever. The result of my observations permits me to state positively that the fever produced by inoculation is attended with no danger, and it is safe to inoculate, as I have already done, from children of two years of age to the oldest individuals. Many of the persons inoculated have come to this city, and in no case has the yellow fever attacked them, which gives me hope of a final result completely satisfactory. The municipality, assisted by the merchants sent to Mexico, January 10, a commission composed of two physicians, in order to study the inocculation of the fever."—*Med. and Sur. Reporter.*

**The Etiology of Dysentery.**—In a communication to the Bohemian Medical Society Prof. Hlava gives an account of a number of observations he has made on the subject of the etiology of dysentery. He examined the stools and the bowels with their contents post mortem in sixty cases of epidemic dysentery, also in two cases of a sporadic nature, and in ten where the symptoms of dysentery were developed as complications of other diseases. He also injected the fresh stools of dysenteric patients into the rectum or duodenum in seventeen dogs, six cats, eight rabbits, hens, and porpoises, and made seventy cultivations of bacteria of various kinds found in dysenteric stools. The conclusion he arrived at was that bacteria are not the cause of dysentery, for none of the micro-organisms were reproduced in the animals experimented upon, only a transitory catarrh being found. No qualitative anatomical distinction could be made out between cases of epidemic and of sporadic origin. Prof. Hlava investigated the amœbiform, large-grained protoplasm which he found in the dejections in considerable quantity in sixty-five cases, and on the intestinal mucous membrane and in the submucous tissue in twenty cases. On putting these bodies under the microscope, he found that the amœboid movements continued for ten hours. He has commenced a series of observations on the effects of injecting them into the intestines of animals, with the object of showing that these bodies have a causal relation to the disease.—*Medical and Surgical Reporter.*

**The Use of Spleen-Pulp in Anæmia.**—Maragliano has reported the results of his experiments with spleen-pulp in cases of impoverished blood and the conditions depending upon it. The pulp was given with an emulsion of bitter almonds in accordance with the following formula:

℞. Emuls. amygd., amar.............................℥ x.

Pulpæ splenic..................................℥ iv,

Spt. vini gallici (cognac)........................℥ ii.

This amount was given in twenty-four hours with meals. The diet consisted of soup with rice, or bread, at eight o'clock in the morning; a second soup, with four ounces of meat and bread, with a glass of wine, at noon; and a third soup at six in the evening. Five cases of chloroses were treated in this manner, with the result, in from eight to twenty-five days, as follows: increase of general strength, increase of red blood-corpuscles; rapidly restored functional activity of the nervous, digestive, circulatory, and urinary mechanism; increase of arterial tension and body-weight. The number of cases is manifestly too small for accurate results, but sufficiently large to awaken interest in the mode of treatment.—*Deutsche Medizinial Zietung.*

**A writer** in the *Mass. Med. Journal* recommends for burns penciling with equal parts of sweet oil and white of eggs, and then applying an ointment composed of one drachm finely powdered alum, two ounces sweet oil, and a teacupful of fresh lard.—*Medical World.*

**Drumin.**—*Rundschau* (Prag) states that Reid isolated an alkaloid from *Euphorbia drumondii*, Boiss., growing in Australia, which he named drumin. The anæsthetic properties of drumin are almost identical with those of cocaine.

